# Dealing with Trade-Offs in Destructive Sampling Designs for Occupancy Surveys

**DOI:** 10.1371/journal.pone.0120340

**Published:** 2015-03-11

**Authors:** Stefano Canessa, Geoffrey W. Heard, Peter Robertson, Ian R. K. Sluiter

**Affiliations:** 1 School of BioSciences, University of Melbourne, Victoria, Australia; 2 School of Science, Information Technology and Engineering, Federation University, Ballarat, Victoria, Australia; University of Colorado, UNITED STATES

## Abstract

Occupancy surveys should be designed to minimise false absences. This is commonly achieved by increasing replication or increasing the efficiency of surveys. In the case of destructive sampling designs, in which searches of individual microhabitats represent the repeat surveys, minimising false absences leads to an inherent trade-off. Surveyors can sample more low quality microhabitats, bearing the resultant financial costs and producing wider-spread impacts, or they can target high quality microhabitats were the focal species is more likely to be found and risk more severe impacts on local habitat quality. We show how this trade-off can be solved with a decision-theoretic approach, using the Millewa Skink *Hemiergis millewae* from southern Australia as a case study. *Hemiergis millewae* is an endangered reptile that is best detected using destructive sampling of grass hummocks. Within sites that were known to be occupied by *H*. *millewae*, logistic regression modelling revealed that lizards were more frequently detected in large hummocks. If this model is an accurate representation of the detection process, searching large hummocks is more efficient and requires less replication, but this strategy also entails destruction of the best microhabitats for the species. We developed an optimisation tool to calculate the minimum combination of the number and size of hummocks to search to achieve a given cumulative probability of detecting the species at a site, incorporating weights to reflect the sensitivity of the results to a surveyor’s priorities. The optimisation showed that placing high weight on minimising volume necessitates impractical replication, whereas placing high weight on minimising replication requires searching very large hummocks which are less common and may be vital for *H*. *millewae*. While destructive sampling methods are sometimes necessary, surveyors must be conscious of the ecological impacts of these methods. This study provides a simple tool for identifying sampling strategies that minimise those impacts.

## Introduction

Occupancy surveys are fundamental to mapping and monitoring species distributions [1], as well as habitat modelling [2], systematic conservation planning [3] and environmental impact assessments [4,5]. In their simplest application, occupancy surveys provide strict assessments of the presence or absence of a species at a given locality; the species is present if it is detected and absent if it is not. However, this intuitive result is confounded by the fact that species may go undetected during a survey, producing a “false absence” [6,7,8]. False absences can lead to biased estimates of the probability of occupancy [9,10], and may undermine the application of survey results.

The reliability of occupancy surveys therefore depends on achieving a sufficiently high probability of detecting the target species if it is present [6,7]. Estimates of the probability of detection during a single survey can be derived using occupancy models, which jointly estimate the probability of site occupancy [5,11,12]. In turn, one can estimate the number of surveys needed to increase the cumulative probability of detection at each site to some desired threshold [13,14]. However, the number of surveys also influences the overall size and cost of a survey program. Hence, in addition to the primary objective of attaining a desired cumulative probability of detection, surveyors will also strive to minimise replication. Several examples exist of how to approach this problem from an economic perspective [5,13,15]. However, certain survey types also have important ecological costs which need to be considered in survey design. Destructive sampling of favoured microhabitats is an example.

Destructive sampling techniques are employed to detect cryptic animals that cannot be readily observed or trapped. Occupancy surveys that use destructive sampling entail searching and destroying favoured microhabitats of the focal species within a given site; in which case searches of multiple microhabitats may be considered equivalent to repeated surveys at each site ([16], p. 162). Examples of destructive sampling include raking beds of leaf litter when searching for fossorial lizards [17], prizing open or lifting (and therein destroying) decaying woody cover when sampling salamanders [18,19], removing exfoliating bark from trees when searching for arboreal arthropods [20] and drag-netting beds of aquatic vegetation for fish or amphibian larvae [18,21]. If microhabitats do not vary in quality for the target species, then the probability of detection at each microhabitat will not vary, and minimising the impacts of destructive sampling is equivalent to minimising the number of microhabitats searched. However, microhabitats usually vary in quality, in which case the focal species is more likely to utilise (and be detected in) some microhabitats than others. In turn, this produces a trade-off in destructive sampling designs, between minimising the loss of high quality microhabitats and minimising replication. To minimise replication, the most effective approach is to sample the highest quality microhabitats, because this confers a higher probability of detection per sampling unit. However, this would also lead to the destruction of the highest quality microhabitats for the target species. Conversely, one could limit sampling to lower quality microhabitats, but the resulting increase in replication may come at considerable financial cost and produce wider-spread impacts on the focal species.

This combination of objectives—attaining a threshold cumulative probability of detection whilst balancing sampling replication and impacts on high quality microhabitats—leads to an optimisation problem that we believe has not previously been solved. Here we show that when a model of the sampling process for a given species is available, it is possible to use a simple decision-theoretic approach to solve this trade-off in destructive sampling designs. We demonstrate this approach using the design of surveys for the Millewa Skink *Hemiergis millewae* Coventry, a locally endangered lizard from southern Australia [22].

## Methods

### Case study and field surveys

In the state of Victoria, *Hemiergis millewae* is recognized as critically endangered, occurring only in the semi-arid Mallee vegetation of the far north-west [23]. *Hemiergis millewae* inhabits hummocks of *Triodia scariosa* (‘Spinifex’), and the most effective means of surveying for this species is to rake and dismantle individual *Triodia* hummocks [24]. While this ensures that individual lizards are found if they occupy a hummock, it entails destruction of the hummock and possibly a reduction in the habitat suitability of the site for the species. Hence, while further surveys are required to ascertain the conservation requirements of *H*. *millewae* in Victoria [23,24], it is important to minimise the impacts of these surveys on the species.

Two of us (PR and IS) conducted surveys for *H*. *millewae* at 52 sites across the Murray-Sunset National Park in north-western Victoria in the Austral autumn of 2011 to improve knowledge of the distribution of this species [24]. Sites were rectangular quadrats measuring 50 m by 20 m, each including multiple *Triodia* hummocks. A variable number of these hummocks were searched at each site, dependent on hummock density and when and if *H*. *millewae* was found (surveys were terminated as soon as an individual was detected). Each hummock was methodically dismantled and searched for individual lizards. Sand and litter beneath the hummock were also gently raked for lizards sheltering therein. The dimensions of each *Triodia* hummock were measured, and hummock volume (m^3^) estimated by assuming a standard rectangular shape. The growth phase of each hummock was also recorded as: 1 = seedling, 2 = immature clump, 3 = mature clump, 4 = mature clump with central tillers beginning to collapse, 5 = central tillers collapsed forming a broken or unbroken ring. Distance to the nearest hummock and the leaf litter cover around each hummock were also measured. All survey work undertaken during this study was carried out in accordance with the requirements of animal ethics and research permits (ethics approval n. 22–08, issued by the Wildlife and Small Institutions Animal Ethics Committee of the Department of Primary Industries; research permit n. 10004684, issued by Department of Sustainability and Environment in accord with Wildlife Act 1975 and National Parks Act 1975).

### Statistical analysis

Initially, we sought to model the probabilities of site occupancy and detection of *H*. *millewae* through the use of a standard occupancy model [11], using individual hummocks within a site as replicate surveys, and seeking relationships between hummock characteristics and the probability of detection on a per hummock basis. However, the data were insufficient to separate the probability of site occupancy and detection, and hence, to gain estimates of the effects of individual hummock properties on the probability of detection. Therefore, we assessed the influence of hummock attributes on the detection of *H*. *millewae* using data from the subset of sites at which this species was observed at least once (19 sites and 85 hummocks), in which case occupancy of these sites by the species was certain (following [7,25]). Candidate logistic regression models were fitted to the hummock-level detection data from known occupied sites as follows:

$$\begin{array}{l} \text{logit}\left(p_{i}\right)=\alpha +\beta X_{i},\\ Y_{i}∼Bernoulli\left(p_{i}\right) \end{array}$$ Eqn 1

where *Y*_i_ is the detection or non-detection of *H*. *millewae* in hummock *i*, represented as a Bernoulli variable with probability *p*_*i*_, which is a logistic function of hummock attribute *X*_*i*_. Due to sample size limitations, additive combinations of hummock attributes were not assessed. We also fitted a “null” model with constant *p*. This led to a candidate set of five single-variable models (null, hummock volume, hummock growth phase, distance to nearest hummock and surrounding leaf litter). Treating detections in different hummocks as independent was justified by the fact that detections were not spatially correlated (Moran I statistic standard deviate = -0.397, *p*-value = 0.654).

The relative fit of these models to the data was assessed using the deviance information criterion (DIC: [26]). DIC balances the unexplained variance in the model and the number of parameters. The model with the lowest DIC value (DIC_min_) is considered the most parsimonious, and models with ΔDIC < 2 (ΔDIC = DIC—DIC_min_) are considered largely indistinguishable. Model fitting was completed using JAGS [27], with uninformative priors for all parameters (code and data provided in S1 Code and S1 Dataset). For each model we ran 100,000 iterations on three Markov chains, after discarding the first 50,000 iterations as a burn-in. The model with the greatest support was used to estimate the cumulative probability of detection (*P*) at a site after surveying *n* hummocks, as [7]:

$$P=1-\prod_{i=1}^{n}\left(1-p_{i}\right)$$ Eqn 2

where *p*_*i*_ is the probability of detection at hummock *i* as given by Eqn 1.

Equation 2 shows that *P* increases with *n*; however, when *p*_*i*_ depends on the characteristics of the hummocks searched, as per Eqn 1, it is also possible to increase *P* by selectively searching particularly suitable hummocks. As above, the decision about which parameter to manipulate depends on the relative importance given to the number or the quality of the hummocks searched. We explored how the optimal survey program varied depending on this importance. A weighting (*w*_*n*_) of between 0 and 1 was assigned to the alternative objectives of minimising the number of hummocks sampled and minimising the quality of hummocks searched, with the total weight summing to one. We combined the two variables influencing *P* (*n*: number of hummocks searched; *X*: predictor of the quality of each hummock searched) into a single objective function of aggregate impact (*A*) to be minimised. This function differs slightly depending on the relationship between detection and the predictor of hummock (microhabitat) quality. When the relationship is positive, the aggregate impact *A* can be calculated as:

$$A=\frac{n-\min(n)}{\max(n)-\text{min(}n\text{)}}\times w_{n}+\frac{X-\min(X)}{\max(X)-\min(X)}\times (1-w_{n})$$ Eqn 3

where *n* and *X* are each rescaled to range between 0 and 1 (by subtracting the minimum value observed during field surveys and dividing by the observed range), and *w*_*n*_ indicates the weight on replication and 1- *w*_*n*_ the weight on microhabitat quality. In the event of a negative relationship between detection and the microhabitat-related predictor, Eqn 3 can be reformulated as:

$$A=\frac{n-\min(n)}{\max(n)-\min(n)}\times w_{n}+\frac{\max(X)-X}{\max(X)-\min(X)}\times (1-w_{n})$$ Eqn 4

We used the Solver add-in in MS Excel to minimise the value of *A* by finding the optimal combination of *n* and *X* (assuming all *n* surveyed hummocks have quality *X* or better). To reflect the influence of *n* and *X* on the probability of detection, we replaced *p*_*i*_ in Eqn 2 with the back-transformed logistic expression from Eqn 1 (using the mean estimated parameters), and set the resulting *P* as a constraint of the optimisation. We carried out the analysis for a target of *P* = 0.95 across all possible weights on hummock number and quality. We also set the minimum and maximum values for *n* and *X* observed in the field as constraints to the optimisation, to prevent the optimal strategy from entailing unrealistic or impractical values of *n* and *X*. The spread sheet for the optimisation is provided in S1 Spreadsheet.

### Sensitivity analysis

In the procedure described above, we chose to subset the data and only analyse detections from sites that were known to be occupied; however, some of the sites where *H*. *millewae* was not observed might in fact have been occupied. If so, the regression parameters in Eqn 1 could be overestimates of the true relationship between hummock characteristics and the probability of detection. Therefore, we repeated all analyses using the full dataset, which represents the other end of the uncertainty spectrum (the possibility that all sites where the species was not detected were in fact occupied, and hence, the full data provides accurate estimates of the effects of hummock characteristics on the probability of detection). We repeated the model selection procedure and obtained estimates for Eqn 1 from the model with the highest DIC support. We then re-evaluated the optimal survey protocol (as per Eqn 3) using the hummock-detection relationship estimated from the entire dataset.

## Results

**Fig 1:**
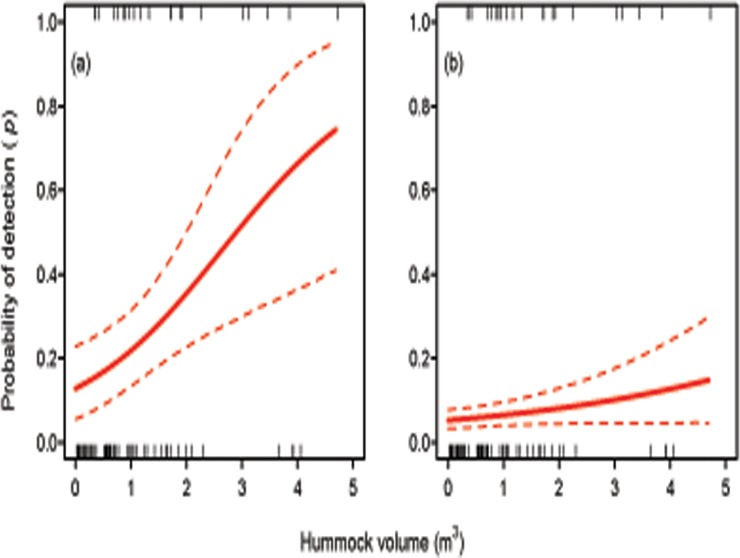
Relationship between the volume of a hummock and the probability of detecting *Hemiergis millewae* in that hummock. Panel (a) depicts the relationship estimated from sites with known occupancy only; panel (b) depicts the relationship estimated from the full set of surveyed sites. Dashed lines represent 95% credible intervals. Inner tick marks display the volume of hummocks in which *H*. *millewae* was detected (top) or not detected (bottom).

Within the subset of sites that were known to be occupied by *H*. *millewae*, the model that included hummock volume as a predictor of the probability of detection (*p*_*i*_) received the most support (DIC = 88.4). The second-best model, including hummock stage, received effectively no support (ΔDIC = 7.8). The probability of detection in a given hummock increased linearly with its volume (Fig. 1a). As expected, results changed when we modelled hummock detection data from all sites. The model including hummock volume still showed the highest level of support based on DIC (the null model being second, with ΔDIC = 1.4). However, the estimated relationship between hummock volume and skink detection was less markedly positive (Fig. 1b).

**Fig 2:**
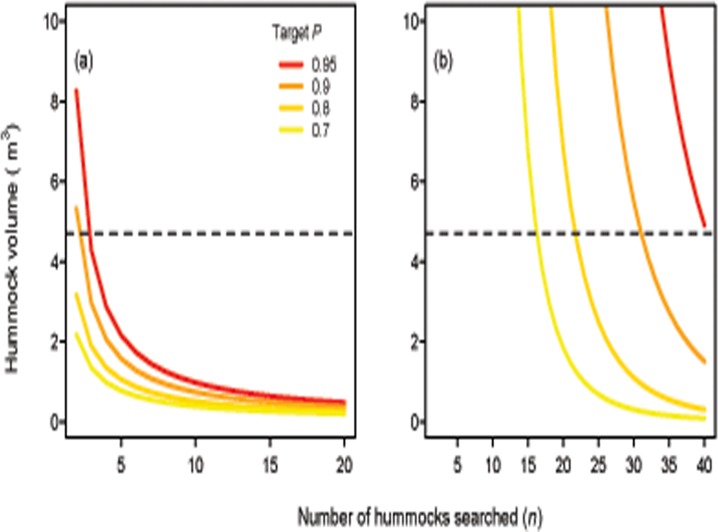
Relationship between search strategies and the cumulative probability of detection of *Hemiergis millewae* at a site. The solid lines show the number of *Triodia* hummocks that must be searched at each site to achieve a given cumulative probability of detection (*P*) of *H*. *millewae*, dependent on the minimum volume of each hummock searched. Panel (a) depicts the relationship using the detection model derived from sites with known occupancy only; panel (b) depicts the relationship using the detection model derived from the full set of surveyed sites. Contour lines depict this relationship for four different values of *P*: 0.95, 0.9, 0.8 and 0.7 (top to bottom). The dashed horizontal line indicates the maximum hummock volume recorded in the model data.

Fig. 2a depicts the trade-off between the number and size of hummocks that must be sampled to achieve a threshold cumulative probability of detection (*P*) using the relationship between hummock volume and the probability of detection estimated from known occupied sites. In general, the minimum volume of hummocks searched needed to increase considerably when searching less than five hummocks (Fig. 2a). When the probability of detection was estimated using the full dataset, the cumulative probability of detection depended almost exclusively on replication, as detection would increase appreciably only under unrealistic values of hummock volume (>10 m^3^). In turn, high targets for the cumulative probability of detection could only be achieved with very large amounts of replication, even for the maximum value of hummock volume observed in the field (*n* = 17 for *P* = 0.7, *n* = 22 for *P* = 0.8, *n* = 31 for *P* = 0.9 and *n* = 45 for *P* = 0.95; Fig. 2b).

**Fig 3:**
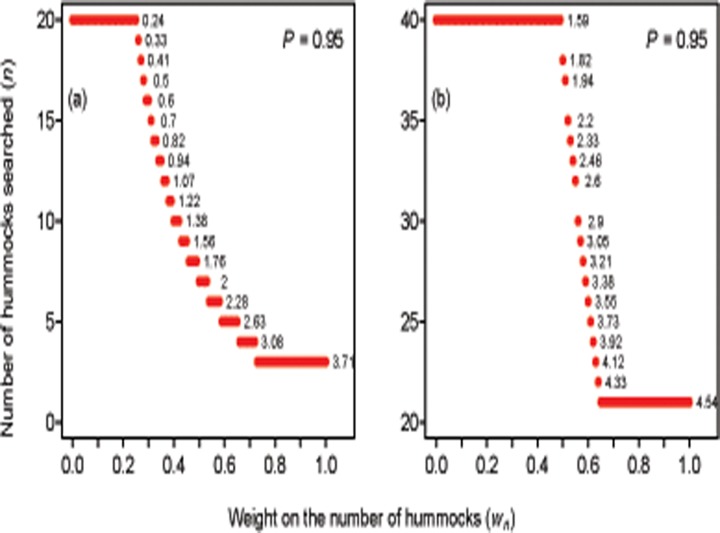
Optimal search strategies for *Hemiergis millewae*. The plot depicts the minimum number of *Triodia* hummocks that must be searched at each site to reach a cumulative probability of detection of *H*. *millewae* of 0.95, dependent on the minimum volume of each hummock searched and the weight (importance) given to either minimising replication or the quality of hummocks searched. The x-axis displays the weight on minimising the number of hummocks sampled (*w*_*n*_). The weight on minimising the volume of each hummock searched is 1 minus *w*_*n*_. Hence, a value of 0.5 on the x-axis corresponds to placing equal weight on minimising the number of hummocks sampled and minimising their quality. The text to the right of each combination shows the minimum volume of the hummocks to be searched to achieve the threshold cumulative probability of detection of 0.95. Panel (a) indicates the optimal strategies based on the detection model derived from sites with known occupancy only; panel (b) describes the optimal strategies based on the detection model derived from the full set of surveyed sites.

Fig. 3a depicts the minimum combination of the number and volume of hummocks that must be searched at a site to reach a cumulative probability of detecting *H*. *millewae* of 0.95 while minimising aggregate impact, dependent on the weight given to minimising the number of hummocks searched per site (*w*_*n*_), and assuming the relationship between hummock volume and detection estimated from the subset of sites that were known to be occupied. Placing high weight on reducing the volume of hummocks searched (low *w*_*n*_) necessitated high sampling replication. For *P* = 0.95 and *w*_*n*_ between 0 and 0.25, this was equivalent to the upper constraint we set in the optimisation problem (*n* = 20). Only two of the 52 sites surveyed during this study received an equivalent level of replication, suggesting that this level of replication may not be practical in many situations. The number of hummocks to be sampled fell exponentially as *w*_*n*_ increased once a particular threshold of this weight was crossed (*w*_*n*_ = 0.26). However, when high weight was placed on minimising replication (*w*_*n*_ ≥ 0.66) the optimal strategy required very large hummocks to be searched. For example, *P* = 0.95 could be achieved by searching three very large hummocks (3.71 m^3^) at a site (Fig. 3b), but only six hummocks in the training data were this size or greater (7%). These results reflect the constraints we chose for the optimisation: in this sense, the optimisation not only identifies the minimum number and size of hummocks to search given differing weights on these two criteria, but can also indicate how practical those weightings are for real-world field surveys.

For the case in which the probability of detection per hummock was estimated from the full dataset, no detection target above 0.7 could be achieved under the original constraint of *n* ≤ 20 hummocks searched per site, as this would require unrealistic values of hummock volume (as above). Removing this constraint allows optimal strategies to be calculated, but they were likely to be impractical (Fig. 3b). For example, a detection target of 0.95 could only be reached by sampling at least 40 hummocks of 1.59 m^3^ (Fig. 3b); equating to 42 hummocks of above average size (the mean observed in the field was 1.12 m^3^).

## Discussion

Our method provides a simple solution to the trade-off implicit in destructive sampling designs for occupancy surveys as exemplified by the case of *H*. *millewae*. Our results suggest that larger *Triodia* hummocks are preferentially used by *H*. *millewae*, and may be an important microhabitat for this species. Yet, if this is true, dismantling large hummocks is also the most effective survey technique for this species. Hence, when designing occupancy surveys for *H*. *millewae*, surveyors face a dilemma: they need to reduce the probability of false absences to an acceptable level, but must also minimise the number and quality of hummocks sampled. Our optimisation approach can be used to identify survey strategies that solve this trade-off, dependent on the importance surveyors give to the number and quality of the microhabitats that will be affected at each site.

When the relationship between hummock characteristics and the probability of detection was estimated from known occupied sites, the trade-off in our case study became especially important if high weight was placed on minimising the number of hummocks to be sampled per site (*w*_*n*_ ≥ 0.66). Under this constraint, the number of hummocks to sample at each site was small (three), but the size of these hummocks needed to be large (≥ 3.71 m^3^) to attain a cumulative probability of detection of 0.95. As above, hummocks of this size were rare in the field data (7%). Hence, targeting hummocks of this size entails the removal of a locally scarce and potentially important resource for *H*. *millewae*. Since lower threshold values of *P* would require less searching effort, they would also entail less overall impacts; however, even moderate detection targets and weight on minimising the number of hummocks sampled could lead to impacts of some magnitude on the target species. For example, re-running our analysis with *P* = 0.8 and *w*_*n*_ = 0.4 indicated that five hummocks of at least 1.5 m^3^ would need to be destroyed to attain the detection threshold. Less than one-third of hummocks in our training dataset were equal to or greater than this size, indicating that they are also a relatively uncommon and potentially important resource. Selecting very large hummocks can also create further problems: here, we interpret spatial sub-units within a site (hummocks) as temporal replicates. Destructive sampling necessarily occurs without replacement, and could generate bias due to the dependency between samples [28]. This bias may become more severe as the ‘population’ (the subset of hummocks with the desired characteristics) becomes smaller.

Considerations such as these are fundamental to setting the weighting scheme in our approach. Ideally, the weight given to either minimising replication or minimising the quality of microhabitats sampled would reflect information on the impact of different sampling protocols on the target species. Where available, occupancy or population models could be used to establish the effect of microhabitat removal on population trends (see for example [19,29]), and weights derived based on the sensitivity of the species to microhabitat loss. One could even extend the approach presented here to explicitly take the modelled effect of microhabitat loss on population trends into account. For example, the decrease in occupancy or population size that would result from a given sampling strategy (i.e., combination of *n* and *X*) could be used as the basis for defining *A* (the aggregate impact) in the objective function (Eqn 3). The aim would remain to minimise *A* using Eqn 3 whilst maintaining the desired value of *P*, because both would be determined by the values of *n* and *X* (*P* through Eqn 1 and 2, and *A* through the occupancy or population model). As above, our data were insufficient to model site occupancy by *H*. *millewae* while accounting for imperfect detection. However, should subsequent studies enable the relationship between the probability of site occupancy and site hummock attributes to be estimated, the change in the probability of site occupancy that would result from a given survey strategy (based on the reduction in the density and volume of hummocks it would cause) could be considered directly in the objective function.

Nevertheless, obtaining quantitative estimates of the impacts of particular sampling regimes may not be possible in many situations. Our simple weighting scheme allows expert judgement on the sensitivity of the target species to destructive sampling to be incorporated into the sampling design. With an appropriate experimental design, adaptive management approaches [30] could be used to learn about the actual impacts of different sampling regimes and update the subjective weightings. Practical aspects of sampling will also influence the range of feasible survey strategies. In our case study, we set upper thresholds for the number and volume of hummocks to search based on the values observed in the field. Moreover, as we have shown here, one can reference the optimal combination of the number and quality of microhabitats to search for a given weighting scheme back to data on the density and quality of microhabitats that are available in the field, to assess the practicality of each weighting scheme. Our simple method therefore also allows the realities of sampling to be accounted for when identifying the optimal sampling regime. We view this as a particularly useful feature of the optimisation approach presented here.

Uncertainty will also surround the estimates of the relationship between the features of microhabitats and detection of the target species. A common cause of uncertainty will be the scarcity of existing data. In our case study, this problem led to us being unable to fit a standard occupancy model to the data. We chose instead to model the effect of microhabitat characteristics on the probability of detection using data from sites with known occupancy; however, this might have led to overestimates of the true microhabitat-detection relationship. If so, the recommended optimal strategy may be insufficient to meet the target cumulative probability of detection. On the other hand, if some or all of the sites where the species was not detected were truly unoccupied, using all available data might produce underestimates of the microhabitat-detection relationship. In this case, the cumulative probability of detection given by the recommended optimal strategy may exceed the target probability; and a strategy with a lower aggregate impact could have been chosen instead.

The effects of uncertainty will be higher when heavy emphasis is placed on minimising replication or minimising impacts on high quality microhabitats, because, as revealed by our optimisations, extreme weightings lead to extreme sampling regimes (very high replication or very high quality of microhabitats to sample). Surveyors should ideally assess the ramifications of uncertainty in the microhabitat-detection relationship for the optimal sampling strategy. In a Bayesian context, investigators can do so by repeating the optimisation when sampling randomly from the posterior distribution of the parameters of the detection model. Our spreadsheet can be used to run simulations of this kind, by sampling at random from specified parameter distributions for the coefficients of the detection model instead of entering fixed values. One can then identify the optimal search strategy for each combination of parameter estimates, and obtain a distribution of optimal strategies which reflects parametric uncertainty. Several add-ins to MS Excel such as MCSimSolver (http://www3.wabash.edu/econometrics/EconometricsBook/Basic%20Tools/ExcelAddIns/MCSimSolver.htm) can be used to run such simulations.

In this study, we limited our scope to the impacts of hummock destruction on the target species. However, destructive sampling for *H*. *millewae* will impact, to some degree, numerous co-occurring species in Mallee environments that utilise *Triodia* hummocks [23,31,32,33]. Impacts on co-occurring species may be an important consideration for destructive sampling designs in general. These additional impacts can be accounted for using the approach we have presented here. As for the single-species case, the weighting scheme could be set using expert opinion on the impact of sampling on other species, or quantitative estimates of the ecological impact of particular sampling designs could be incorporated directly into the objective function.

Non-destructive sampling methods are always preferable, but destructive searches are necessary for some species. Yet studies that rely on such methods run the risk of undermining their very purpose, by negatively impacting the focal species or community [19]. When destructive methods are necessary, practitioners should carefully consider the trade-off between minimising replication and minimising the destruction of high quality microhabitats. The method we have presented provides a simple quantitative tool for assessing this trade-off.

## Supporting Information

S1 Code JAGS code for the detection model for *Hemiergis millewae*. (PDF)

S1 Dataset Data for the detection model. (TXT)

S1 Spreadsheet Spreadsheet for optimisation of search strategies. (XLSM)
