# Supplementary material for: Dealing with Trade-Offs in Destructive Sampling Designs for Occupancy Surveys
Source: PLoS One. 2015 Mar 11;10(3):e0120340. doi: 10.1371/journal.pone.0120340 (PMC4356509; doi:10.1371/journal.pone.0120340)
Supplement: S1 Code — (PDF) [file pone.0120340.s001.pdf]

Logistic model for hummock-level detection of Hemiergis millewae

This code uses JAGS (http://mcmc-jags.sourceforge.net/) to fit logistic regression models to the detection of Millewa skink Hemiergis millewae in spinifex hummocks. We calculate the deviance information criterion of the model, for ranking purposes, and estimate the relationship between detection and microhabitat characteristics (here hummock volume). The estimates of the logistic regression equation will then be used in the optimization spreadsheet.

To start the analysis, install another couple of packages and modules required to run this code:

```
install.packages("R2jags")
library(R2jags)
load.module("glm")
load.module("dic")
```

First create the model in .bug format:

```
sink("hummock_skink.bug")
cat("
    model{
    # Likelihood
        for (i in 1:n.sites){
    # Determine presence in a given hummock
            h.skink[i] ~ dbern(h.occ[i])
            logit(h.occ[i]) <- a.h + b.h*predictor[i]
        }

    # PRIORS for the coefficients

    # Intercept
    a.h ~ dnorm(0, 0.001)
    # Slope
    b.h ~ dnorm(0, 0.001)

  # Make predictions of detection
  for (i in 1:470){
  pred_vol[i] <- i*0.01
  logit(p.occ[i]) <- a.h + b.h*pred_vol[i]
  }
}
")
sink()
```

Now load the data and create a list for the model. You will need to set the correct working directory before uploading the data (text file provided).

```
setwd("C:/workdir") # Set your working directory
skink.data <- read.table("Appendix_B_data.txt", header=TRUE)
# Change "predictor" to the variable of interest
skink.data <- list(n.sites=dim(skink.data)[1],
                   predictor=skink.data$h_vol,
                   h.skink=skink.data$skink)
```

Set the initial values for the parameters,

```
skink.inits <- function() {
    list(a.h = rnorm(1), b.h = rnorm(1))
}
```

specify the MCMC simulation settings,

```
ni <- 10000  # iterations
nt <- 5  # thinning rate
na <- 1000  # adaptation phase
nb <- 5000  # burn-in
nc <- 3  # separate chains
```

and specify the parameters to monitor.

```
parameters <- c("a.h", "b.h", "deviance", "p.occ")
```

Now we are ready to run the model using JAGS (make sure you've downloaded and installed JAGS first!).

```
# Adaptation phase
skink.run <- jags.model(data=skink.data, inits=skink.inits,
                        file="hummock_skink.bug",
                        n.chains=nc, n.adapt=na)
# Burn-in
update(skink.run, nb)
# Update samples
skink.samples <- coda.samples(skink.run, variable.names=parameters,
                              n.iter=ni, thin=nt)
# Calculate deviance information criterion (DIC)
skink.DIC <- dic.samples(skink.run, variable.names=parameters,
                         n.iter=ni, thin=nt, type="pD")
```

Now we can extract the estimates for the parameters of the logistic regression: we calculate mean, standard deviation and 95% credible intervals for the distribution of each.

```
skink.samples.mat1 <- as.matrix(skink.samples[[1]])
skink.samples.mat2 <- as.matrix(skink.samples[[2]])
skink.samples.mat3 <- as.matrix(skink.samples[[3]])

posterior.estimates <- rbind(skink.samples.mat1,
                             skink.samples.mat2,
                             skink.samples.mat3)
vars <- t(as.matrix(posterior.estimates))
parameter.mean <- apply(vars, 1, mean)
parameter.sd <- apply(vars, 1, sd)
parameter.lower.ci <- apply(vars, 1, quantile, c(0.025))
parameter.upper.ci <- apply(vars, 1, quantile, c(0.975))

skink.results <- data.frame(mean=parameter.mean,sd=parameter.sd,
                            lowerCI=parameter.lower.ci,
                            upperCI=parameter.upper.ci)
```

We visualize the estimates for the intercept and slope parameters, as well as the DIC for this model:

```
skink.results[1:2, ]
skink.DIC
```

We can export these to a text file for easier comparison.

```
sink("Model_results.txt", append = T)
cat("\n")
cat("########################", "\n")
cat("Model: log(volume)", "\n")
cat("\n")
cat("Regression parameters", "\n")
skink.results[1:2, ]
cat("\n")
cat("Deviance information criterion", "\n")
skink.DIC
cat("\n")
sink()
```
